# Standoff Detection of Uranium and its Isotopes by Femtosecond Filament Laser Ablation Molecular Isotopic Spectrometry

**DOI:** 10.1038/srep43852

**Published:** 2017-03-08

**Authors:** Kyle C. Hartig, Isaac Ghebregziabher, Igor Jovanovic

**Affiliations:** 1 Department of Mechanical and Nuclear Engineering, The Pennsylvania State University, University Park, PA 16802 USA; 2 Department of Nuclear Engineering and Radiological Sciences, University of Michigan, Ann Arbor, MI 48109 USA

## Abstract

The ability to perform not only elementally but also isotopically sensitive detection and analysis at standoff distances is impor-tant for remote sensing applications in diverse ares, such as nuclear nonproliferation, environmental monitoring, geophysics, and planetary science. We demonstrate isotopically sensitive real-time standoff detection of uranium by the use of femtosecond filament-induced laser ablation molecular isotopic spectrometry. A uranium oxide molecular emission isotope shift of 0.05 ± 0.007 nm is reported at 593.6 nm. We implement both spectroscopic and acoustic diagnostics to characterize the properties of uranium plasma generated at different filament-uranium interaction points. The resulting uranium oxide emis-sion exhibits a nearly constant signal-to-background ratio over the length of the filament, unlike the uranium atomic and ionic emission, for which the signal-to-background ratio varies significantly along the filament propagation. This is explained by the different rates of increase of plasma density and uranium oxide density along the filament length resulting from spectral and temporal evolution of the filament along its propagation. The results provide a basis for the optimal use of filaments for standoff detection and analysis of uranium isotopes and indicate the potential of the technique for a wider range of remote sensing applications that require isotopic sensitivity.

Laser-induced breakdown spectroscopy (LIBS) is presently widely used for its *in-situ*, remote, and real-time analysis capabilities^1^,^2^,^3^,^4^. In the LIBS technique, the output of a pulsed high-power laser is focused onto the surface of a sample of interest to generate a luminous micro-plasma. The emitted radiation from the plasma is collected and spectrally resolved. The spectral analysis of the atomic, ionic, or molecular emission reveals the elemental composition of the sample. The types of pulsed lasers most commonly used for LIBS are the Q-switched nanosecond (ns) Nd:YAG lasers and amplified ultrafast laser systems, such as those based on chirped-pulse amplification in Ti:sapphire, which routinely produce high-energy femtosecond (fs) pulses. LIBS is an all-optical technique which offers particular benefits in applications where the sample collection is not possible due to limited access or presence of hazardous environment, or the speed of detection and analysis is paramount^5^,^6^.

LIBS is an attractive method for remote measurements, but one of the challenges that arises is the ability to produce a small laser focal spot size on the sample surface at large distances^7^. Diffraction and optical distortions incurred during propagation through air cause the laser focal spot size to increase as the propagation distance increases. A commensurate increase in the laser power is required to achieve plasma formation on the sample surface. It is possible to decrease the spot size at longer distances by increasing the aperture of the focusing optical element; however, due to the physical and cost constraints, the aperture size is usually limited. Further, even high-quality optics suffer from aberrations that, along with the atmospheric distortions, increasing the laser focal spot size and limiting the intensity that can be produced on the target surface. Currently, remote-LIBS (R-LIBS) measurements employing ns lasers have been demonstrated at distances of up to ~100 m^8^.

In contrast to ns-LIBS, fs-LIBS can take advantage of the complex but favorable nonlinear dynamics of fs laser propagation in air. Self-focusing of an initially collimated fs laser pulse occurs when its peak power exceeds the critical power, *P*_*cr*_ = 3.72*λ*^2^/(8*πn*_0_*n*_2_), where *λ* is the laser wavelength in m, *n*_0_ (unitless) is the linear index of refraction, and *n*_2_ in m^2^/W is the nonlinear index of refraction. In this expression *P*_*cr*_ is in the units of W. An interplay of plasma defocusing and nonlinear self-focusing results in generation and stable propagation of a laser filament with a typical intensity on the order of 10^13^ W/cm^2^ 9,10. A filament has a central core with diameter in the range of 100–200 mm, which is surrounded by an energy reservoir^9^,^11^,^12^. Filaments have been shown to be capable of propagating over distances on the order of kilometers^13^. The range of filament propagation is dependent on the initial laser pulse parameters^14^ and on the characteristics of the external focusing^12^. Further, the characteristics of filament-induced plasma, including its optical emission, are dependent on the filament spatial evolution^15^.

Analytic tools that can perform both elemental and isotopic analysis of samples *in situ*, without any sample preparation, are important for remote sensing applications. Isotopic measurements can be performed by LIBS, as has been demonstrated with H, Li, U, and Pu, for example^16^,^17^,^18^,^19^,^20^. Due to the small atomic isotope shift (typically < 0.02 nm), high resolution spectrometers, operation under rarefied atmospheric conditions, and thousands of laser shots are necessary to resolve the isotope shift. These limitations are not always compatible with remote measurements. In the recently developed technique of Laser Ablation Molecular Isotopic Spectrometry (LAMIS)^21^,^22^,^23^, radiative molecular transitions from diatomic molecules are used to extract the isotopic composition of a sample under ambient atmospheric conditions. A similar method has been demonstrated in earlier work by Niki *et al*.^24^, where rarefied atmospheric conditions were used. The molecules originate either from direct vaporization of the sample surface or through plasma-assisted chemical reactions of the vaporized sample with the ambient atmosphere^21^,^25^,^26^.

In this work, we demonstrate that a technique combining fs filamentation and LAMIS (F2-LAMIS) developed by Hou *et al*.^27^. can be used for real-time, isotopically sensitive standoff detection of uranium at standoff distances (1 m in our experiment), suggesting a significant potential for its use in important nuclear security applications. The identification of uranium oxide molecular emissions from a laser induced plasma and the use of the molecular emission isotope shift for isotopically resolved uranium LIBS measurements is reported. Differences in the variation of the emission intensity along the filament propagation distance were observed for molecular, U II (ionic), and U I (atomic) emissions. However, a nearly constant signal-to-background ratio (SBR) was observed along the filament for the uranium molecular emission, in contrast to U II and U I emission, where distinct maxima of the SBR are observed near the end of the filament. The constant molecular emission SBR indicates that the filament propagation distance does not have a significant effect on the performance in F2-LAMIS measurements.

A simplified schematic of the experimental setup is shown in Fig. 1. The laser used is an ultrafast Ti:sapphire based chirped-pulse amplification system operating at 10 Hz. For our measurements, the pulse energy and pulse duration were set to 8.0 mJ and 42 fs, respectively. The collimated laser beam (20 mm diameter at full-width half-maximum) was loosely focused with a 25.4 mm diameter, *f* = 4.0 m plano-convex lens to create laboratory-scale filaments. Two ~0.25 mm-thick sheet uranium samples were used: depleted uranium (DU, ~0.4% ^235^U) and highly enriched uranium (HEU, ~98% ^235^U). The filament propagation distance was varied by sliding the laser focusing lens mounted on an optical rail. Due to safety requirements established for this experiment which include the operation in sealed environment, the laser pulse was focused through a 3 mm thick optical window placed at the end of the vacuum tube, as shown in Fig. 1. The position of the optical window was varied in steps of 30 cm using variable-length sections of flexible vacuum tubing as the focusing lens was translated for the filament propagation measurements in order to compensate for the changing distance between the focusing lens and window at several discrete points in the measurement. The effect the optical window has on the measured LIBS spectra is discussed in detail in Supplementary Note 1. This constraint in the experimental setup results in the variation of laser intensity at the window as the focusing lens is translated, with the commensurate variation of the contribution of the window to self-phase modulation during filament formation. However, the filament propagation distance effects can still be correlated to the filament axial intensity profile through its direct measurement by acoustic diagnostics. We report the distances relative to the geometric focus of the focusing lens, as shown in Fig. 2.

For the filament propagation distance-dependent measurements, the uranium plasma emission was coupled into an optical fiber using a 55 mm-focal length, 25.4 mm-diameter lens mounted at a 45° angle with respect to the filament axis. For standoff measurements, a 10-cm lens was placed 1 m from the sample, also at an angle of 45° with respect to the filament axis, focusing the collected light into an optical fiber. Both fibers were coupled to a Horiba Jobin-Yvon iHR-550 spectrometer equipped with an Andor iStar 334 T ICCD detector. Ten laser ablation accumulations were acquired for each spectral acquisition following five cleaning ablations, which ensured the removal of a possible thin oxide layer on the sample surface. For standoff measurements, 100 spectra were accumulated following five cleaning shots. The length of the propagating filament was obtained by detecting the acoustic shock wave in air using a sensitive microphone (PCB Piezotronics). The acoustic signal as a function of propagation distance of the filament was then obtained by sliding the focusing lens along an optical rail in steps of 10 cm.

## Results

### Filament acoustic measurement

**Figure 2:**
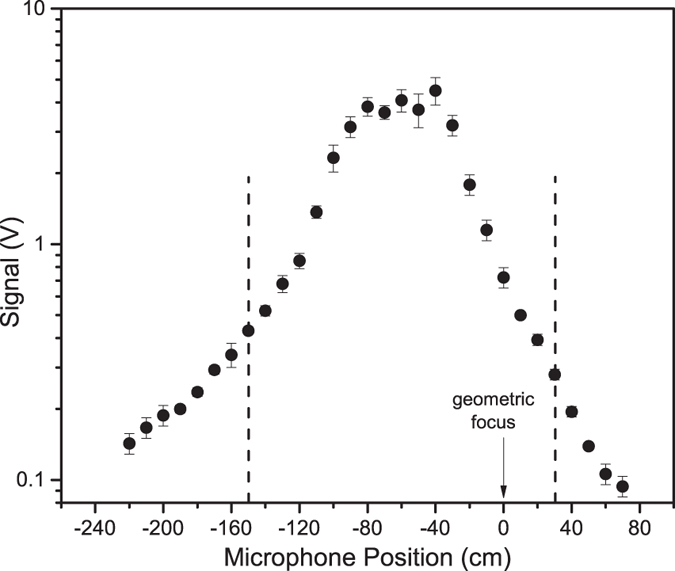
Filament acoustic emission over the filament propagation distance. U II ionic emission (424.43 nm) was observed following filament ablation of the DU sample for distances bounded by the vertical dashed lines.

The measured acoustic signal along the filament propagation is shown in Fig. 2. Acoustic measurements of the shockwave formed by the ionization of air along the propagation of the filament have been previously used to characterize the spatial evolution of the filament intensity^14^,^28^. The filament intensity is related to the free electron density in the filament, and the free electron density can be inferred from the measured acoustic emission of the filament. The filament intensity reaches a maximum 40 cm before the geometric focus of the lens. The length of the resulting filament was determined to be ~3.0 m from acoustic measurements. However, the uranium atomic, ionic and molecular emission were observed only over a 1.8 m-long portion of the filament. The greater filament length determined by the acoustic measurement suggests that at filament-uranium interaction points outside of bounded region shown in Fig. 2, the filament intensity is below the breakdown threshold for uranium. The intensity profile of the filament was measured using burn paper at multiple positions along the filament propagation, confirming the presence of multiple filamentation. The filament-uranium interaction point where the ionic emission is first observed (approximately −150 cm) can be considered the point at which the filament intensity has increased above the breakdown threshold of uranium. Similarly, the interaction point at ~30 cm is interpreted as the point at which the filament intensity drops below the uranium breakdown threshold.

### Filament spectrum measurement

**Figure 3:**
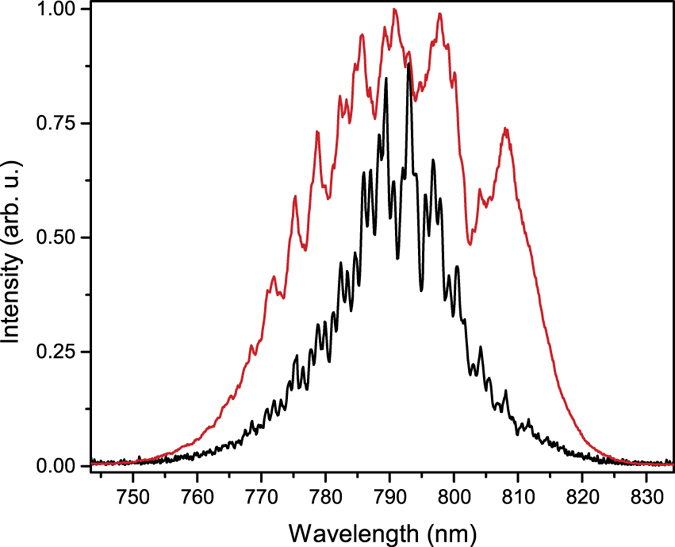
Broadening of filament spectrum. Measured filament spectrum at −120 cm (black) and at −20 cm (red).

As the filament propagates through air and through the optical window, it undergoes significant temporal modulation and spectral broadening, as previously shown in numerical simulations and experimental measurements^29^,^30^. We observed this broadening by inserting an uncoated wedge optic into the filament path at a grazing incidence and coupling the reflected light into a spectrometer. The filament spectrum is shown in Fig. 3 for two filament-uranium interaction points: −120 cm and −20 cm. An aperture was used to partially remove the contribution of the filament reservoir in the measured spectrum.

### Uranium oxide molecular emission and standoff detection

The uranium spectrum between 580 and 600 nm was selected for spectroscopic analysis of the filament-induced plasma, covering the R, Q, and P UO molecular branches^25^,^31^. Previous works by Heaven *et al*. identified the UO molecular emission for U^18^O and U^16^O at low temperatures (130 K)^31^. The authors observed a complex molecular emission structure over the 593.0–593.4 nm spectral region for U^16^O. The higher plasma temperature achieved in these measurements combined with the different ablation and excitation mechanisms results in a single molecular emission line, which is observed at 593.6 nm for the DU sample. The details of the identification of the 593.6 nm emission feature as a uranium molecular emission can be found in Supplementary Note 2. Due to the difference in the shape and position of the molecular emission feature observed in our measurements in comparison to that reported in earlier studies at lower plasma temperatures, the molecular emission can not be identified as UO from these measurements alone, but is assumed to be associated with a uranium oxide (U_*x*_O_*y*_) molecule. The uranium oxide molecular emission was maximized ~500 µm off the surface of the U sample, which is the result of expansion and cooling of the plasma after the 0.35 µs delay used for spectral acquisition.

**Figure 4:**
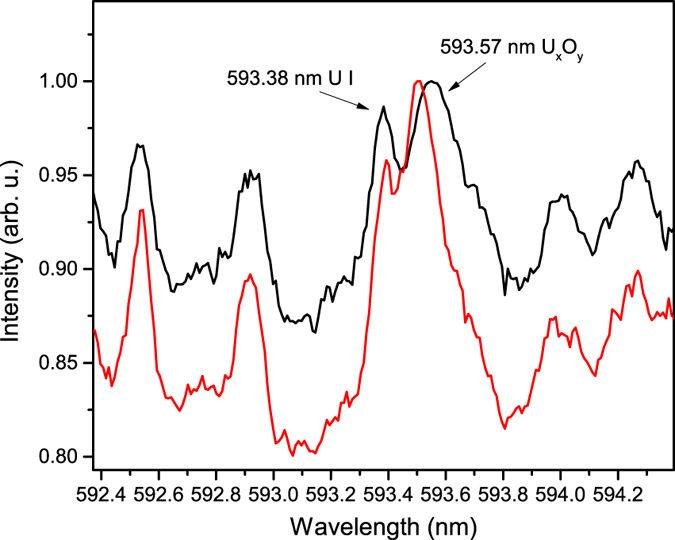
Remote F2-LAMIS U_*x*_O_*y*_ measurement. Standoff detection F2-LAMIS spectra at 1 m of the DU (black) and HEU (red) samples showing a molecular isotope shift of 0.05 nm for the 593.6 nm uranium molecular emission band head.

Standoff detection of DU and HEU was achieved using a 1.2 m long filament and a 1 m detection standoff, which produced an observable molecular isotope shift of 0.05 nm ± 0.007 for the 593.6 nm U_*x*_O_*y*_ molecular emission band head that is shown in Fig. 4, and is approximately a factor of two larger than the largest atomic/ionic isotope shift of 0.025 nm for the U II 424.43 nm emission. Determination of the error associated with the uranium molecular isotope shift measurement is explained in Supplementary Note 3. The gate delay (1.0 µs) and width (10.0 µs) were optimized to maximize the measured molecular emission intensity, which also led to a more robust observation of the molecular isotope shift.

In order to characterize the optical emission of plasmas generated at different filament propagation distances, we studied the intensity variation of the U_*x*_O_*y*_ molecular emission (593.6 nm), the U I atomic emission (591.53 nm), and the U II ionic emission (424.43 nm) using a gate delay and width of 0.35 µs and 5.0 µs, respectively. A Lorentzian line profile was fit to the measured atomic, ionic, and molecular emission lines. The intensity, area, width, and y-offset of the fitted peak were extracted at different filament-uranium interaction points.

### Filament propagation distance resolved uranium plasma emission measurements

**Figure 5:**
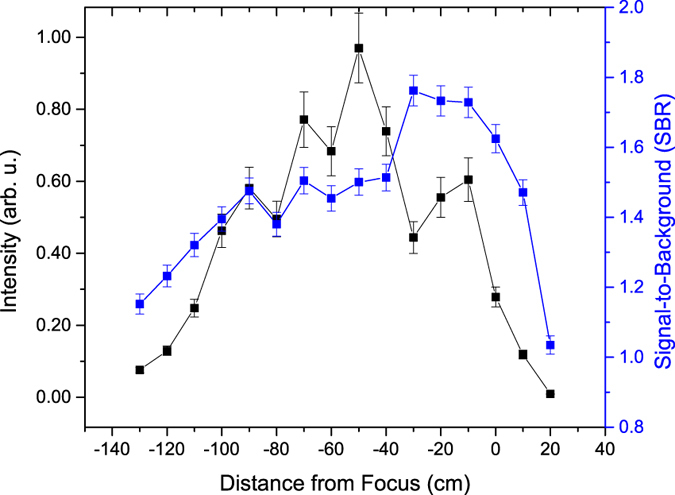
Dependence of uranium molecular emission and SBR on filament propagation distance. The U_*x*_O_*y*_ molecular emission exhibits a nearly constant SBR over the propagation distance of the filament for the DU sample. The peak molecular emission intensity increases over the propagation distance of the filament.

**Figure 6:**
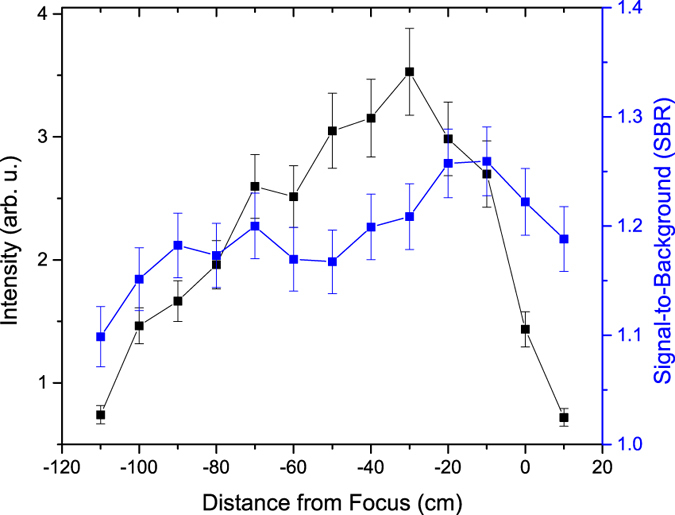
Dependence of uranium atomic emission (591.53 nm) and SBR on filament propagation distance. The atomic emission intensity and SBR increase to their maximum at −50 and −40 cm, respectively for the DU sample. The maximum in the atomic emission at −50 cm indicates that the U density within the plasma is maximum at this filament-uranium interaction point.

**Figure 7:**
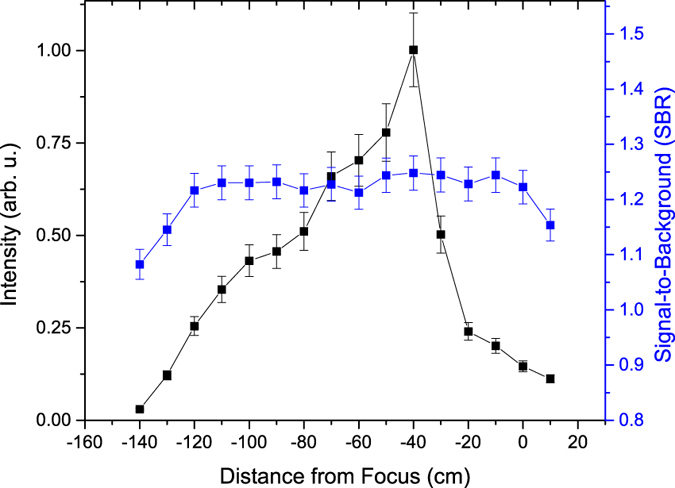
Dependence of uranium ionic emission (424.43 nm) and SBR on filament propagation distance. The intensity and SBR initially remain nearly constant before rapidly increasing to their maximum at −30 and −20 cm, respectively for the DU sample.

The fitted uranium molecular emission intensity and SBR along the propagation distance of the filament are shown in Fig. 5. The intensity of the U_*x*_O_*y*_ emission initially increases with filament propagation distance before reaching a maximum 40 cm before the geometric focus. However, the U_*x*_O_*y*_ SBR (defined as the ratio of the peak intensity of the emission centered at 593.6 nm and the background intensity) initially increased over the first 20 cm of the filament length then maintained a nearly constant value before decreasing over the last 10 cm of the filament length. The uranium atomic (591.53 nm) emission initially increases before reaching a maximum (ten times above the lowest emission intensity) at −50 cm and then rapidly decreases, as shown in Fig. 6. Similarly, the uranium ionic emission (424.43 nm) initially increases before reaching a maximum (five times above the lowest emission intensity) at −30 cm and then rapidly decreases, as shown in Fig. 7. In contrast to the U_*x*_O_*y*_ molecular emission, the SBR of the uranium atomic and ionic emissions rapidly increases to a maximum (~10% and ~50% above the minimum value of the SBR, respectively) 20 cm before the geometric focus and then rapidly decreases.

Variation of the emission intensity along the filament propagation distance depends on the origin of the emission (atomic, ionic, or molecular) and on the characteristics of the plasma. The molecular, atomic, and ionic emission intensities are functions of temperature; however, the molecular emission is dependent on the density of U_*x*_O_*y*_ in the plasma, while the atomic and ionic emission is dependent on the density of U I and U II in the plasma, respectively^21^. Treating the plasma as homogeneous and in local thermodynamic equilibrium (LTE), the dependence of emission intensity on plasma temperature at different filament-uranium interaction points can be calculated. The upper energy level associated with the U II 424.43 nm emission is 3.03 eV^32^. In previous studies by Hou *et al*.^27^ and Harilal *et al*.^33^, the plasma temperature along the filament was shown to vary by Δ*T*/*T* < 10–20%. If a plasma temperature of *T* = 0.8 eV is assumed, a Δ*T*/*T* = 10% change in the temperature results in a Δ*I*/*I* = 30% change in emission intensity. The much greater experimentally measured variation of the U II emission intensity provides evidence that the plasma density also varies along the filament propagation distance.

The maximum in the U_*x*_O_*y*_ emission intensity suggests that at the optimum filament-uranium interaction point (−40 cm) the density of U_*x*_O_*y*_ within the plasma is maximized. In prior profilometry measurements of the filament ablation craters in similar experimental conditions^27^, it has been observed that the greatest mass removal occurs at the peak of the molecular emission. The origin of the molecular emission from a non-oxidized sample has been shown to be due to the formation of diatomic molecules through reactions with the ambient atmosphere^21^,^25^,^27^. The increase of the atomic, ionic, and molecular emission suggests that an increase of the mass of ablated uranium produces an increase in the density of U I, U II, and U_*x*_O_*y*_ within the plasma. From Fig. 6, the U I intensity continuously increases up to −50 cm, which indicates that the density of U in the plasma that could contribute to the formation of U_*x*_O_*y*_ in the plasma increases up to this location. The emission intensity for each uranium emission begins to decrease rapidly at a similar filament propagation distance 30–50 cm before the geometric focus of the lens.

The differences in the SBR profile of the uranium molecular, atomic, and ionic emission along the filament result from the difference in the variation of the plasma density, the U and U_*x*_O_*y*_ density in the plasma, and the background. The background consists of bremsstrahlung and recombination emission and increases at a rate commensurate to that of the U_*x*_O_*y*_ emission. The U I and U II emission, however, increase at a rate greater than the background emission.

Maximization of the ablated mass and the plasma density at the optimum filament-uranium interaction point that leads to an optimization in the SBR for both the atomic and ionic emissions may be a result of the spectral broadening of the filament, which is shown in Fig. 3. The broadened filament spectrum increases the efficiency of multiphoton ionization during the filament-uranium interaction and ablation process. This result agrees well with previous work by Gunaratne *et al*.^34^ that showed a factor of four decrease in the LIBS threshold when the laser bandwidth is doubled from 15 to 30 nm. Further, the broadened filament spectrum may result in a decrease of the pulse duration that could also contribute to the ablation efficiency^9^,^34^. At longer filament propagation distances the filament intensity decreases due to energy depletion.

## Discussion

We have demonstrated the use of F2-LAMIS, a versatile all-optical detection technique, for standoff measurement of uranium with isotopic selectivity. This approach could enable real-time, remote detection of the isotopic composition of multiple materials relevant to nuclear security with no sample preparation. In addition, it could be used for analysis in geophysics and planetary science, as well as environmental monitoring applications where standoff detection, limited sample preparation, and isotope selectivity are desirable. The peak molecular, atomic, and ionic emission were observed at filament propagation distances where the filament intensity was clamped. The increase in the ablation and ionization efficiency at the optimal filament-uranium interaction point could result from the broadening of the filament spectrum due to plasma-induced self-phase modulation and optical Kerr effect, which occurs as the filament propagates through the ambient atmosphere and the optical window^35^. The molecular emission SBR varied by ~10% over most of the filament length due to an increase in the ablated U mass, which leads to formation of U_*x*_O_*y*_ and a commensurate increase in the plasma density. In a recent comprehensive review by Labutin *et al*.^36^ it has been argued that the potential for future use of laser filaments for remote LIBS measurements depends on the continuing advancement of the understanding of the spatial evolution of the laser filament as well as the plasma generation and emission, which this work has partially addressed. In the future it would be beneficial to elucidate the effect the optical window has on the filament formation and evolution compared to the free propagation of the filament. While this work focused on uranium, the propagation distance-dependent results are in good agreement with previous studies of copper by Ghebregziabher *et al*.^15^. Specifically, in the previous work a similar pattern of increase in the emission intensity and SBR for neutral copper at distances before and near (~10 cm) the geometric focus of a forced-focus laboratory-scale filament has been observed. This consistency with results obtained using targets made of different material suggests that the observed optimization of the atomic, ionic, and molecular emission along the filament length could be a more general result.

The measured uranium molecular emission exhibited strong broadening, which caused the individual rotational components of the vibrational emission to merge into a wide band head. The gate delay and width were optimized to concurrently observe the isotope shift and to obtain sufficiently high intensity for remote measurements. The combined use of acoustic and spectroscopic measurements allowed a qualitative relationship between the intensity profile of the laboratory-scale femtosecond filaments and the measured acoustic emission to be established. In the future it will be beneficial to optimize the plasma parameters such that the spectral broadening of the uranium molecular emission can be reduced. While the observed broad molecular emission band head does not prevent discrimination between nearly pure isotopes of uranium, it makes the measurements of intermediate uranium enrichments much more challenging. It was observed that increasing the spectral acquisition gate delay to ≳10 µs reduced the broadening in U_*x*_O_*y*_ molecular emission spectra due to the cooling of the rapidly expanding filament induced uranium plasma. However, this reduction of broadening was accompanied by reduction of the magnitude of the measured U_*x*_O_*y*_ emission. The temporal dependence of the uranium molecular emission is an indication of the dynamics of population of different vibrational and rotational levels involved in the emission. Combined with the lower plasma density and temperature at longer delay times, the gate delay and width could be varied to optimize the measured molecular emission for more robust discrimination of the isotopes of uranium. Lastly, the uranium molecule that results in the observed molecular emission and additional molecular band heads should be identified; however, there is a lack of published work identifying their location. A modeling effort is in progress to identify the location of UO molecular emission band heads, which will assist with their future experimental analysis and characterization.

## Methods

### Radiological safety hazards

Sections of 1 m long steel vacuum tubes and flexible vacuum tubes of variable length were installed onto one port of the vacuum chamber and a 3 mm thick fused silica window was installed on the other end of the tube. This arrangement allows for filamentation to occur within the tube while keeping the entire experiment in sealed environment, per safety and regulatory requirements pertaining to the use of nuclear materials that were established for this experiment. High-efficiency particulate air (HEPA) filters were inserted into vacuum lines attached to the sealed chamber, and three pump-downs of the chamber were carried out to remove any loose contamination before the chamber was opened and the samples were removed. Further, after each experimental campaign the chamber was surveyed using a Ludlum Model 43–2a alpha detector and swipe sample surveys were conducted every month, which were analyzed by liquid scintillation counting. No contamination surveyed in this manner was detected over the duration of the experiment.

### Acoustic measurement

A sensitive microphone (PCB Piezotronics 378A13), was coupled to Stanford Research Instruments SR560 preamplifier and Teledyne LeCroy WaveSurfer 10 oscilloscope to detect the filament-induced acoustic emission. The acoustic signal as a function of the propagation distance of the filament was then obtained by sliding the focusing lens in steps of 10 cm. The peak-to-peak voltage of the first acoustic emission peak produced as the filament propagates through the ambient atmosphere was used for the acoustic emission measurements.

### Sample introduction and spectral analysis

The uranium metal samples were attached to scanning electron microscope (SEM) mounts, which were then mounted to a custom sample holder inside the sample chamber. Gamma spectroscopy was performed on the DU and HEU samples to confirm their ^235^U enrichment of 0.4 ± 0.045% and 96% ± 3.63%, respectively. A Canberra liquid nitrogen cooled LEGe detector with a Canberra Inspector 2000 multi-channel analyzer and Genie 2000 software along with the Multi-group Uranium Analysis (MGAU Version 4.2) software package were used to carry out the uranium enrichment measurements. Each F2-LAMIS spectral acquisition was acquired under atmospheric conditions at room temperature inside the sample chamber and averaged over 10 laser shots to minimize the effects of fluctuations in the laser intensity, plasma formation, plasma optical emission, and electronic noise on the measured F2-LAMIS spectra. Due to the presence of an oxide layer on the samples, the samples were ablated by five laser pulses before each spectral acquisition. These five laser shots served as “cleaning” shots and removed the thin layer of oxidation on the uranium surface before the spectral acquisition. The uranium sample was translated 500 µm after each spectral accumulation using a custom designed stage with three Newport Picomotor piezo linear actuators.

## Additional Information

**How to cite this article**: Hartig, K. C. *et al*. Standoff Detection of Uranium and its Isotopes by Femtosecond Filament Laser Ablation Molecular Isotopic Spectrometry. *Sci. Rep.* **7**, 43852; doi: 10.1038/srep43852 (2017).

**Publisher's note:** Springer Nature remains neutral with regard to jurisdictional claims in published maps and institutional affiliations.

## Supplementary Material

Supplementary Information

## Figures and Tables

**Figure 1:**
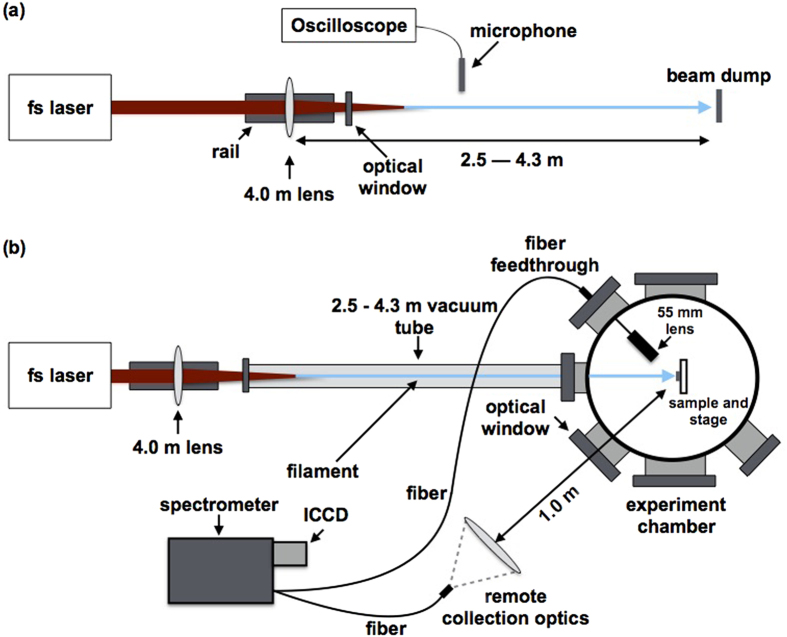
Acoustic measurement and F2-LAMIS setup. (a) Filament propagation distance resolved acoustic measurement setup. (b) F2-LAMIS spectral measurement setup includes both a remote collection using a fiber-coupled lens placed 1 m from the sample and a high-efficiency short focal length fiber-coupled collection optic placed 55 mm from the sample surface.
