# Supplementary material for: Standoff Detection of Uranium and its Isotopes by Femtosecond Filament Laser Ablation Molecular Isotopic Spectrometry
Source: Sci Rep. 2017 Mar 8;7:43852. doi: 10.1038/srep43852 (PMC5341042; doi:10.1038/srep43852)
Supplement: Supplementary Information [file srep43852-s1.pdf]

Standoff Detection of Uranium and its Isotopes by Femtosecond Filament Laser Ablation Molecular Isotopic Spectrometry

Supplementary Information

Kyle C. Hartig[1,†,*], Isaac Ghebregziabher[1], and Igor Jovanovic[1,2]

[1]Department of Mechanical and Nuclear Engineering,
The Pennsylvania State University, University Park, PA 16802, USA
[2]Department of Nuclear Engineering and Radiological Sciences,
University of Michigan, Ann Arbor, MI 48109, USA
[†]Present Address: Department of Material Science and Engineering,
University of Florida, Gainesville, FL 32611 USA
[*]hartig@mse.ufl.edu

Supplementary Note 1: Effect of translation of the optical window on LIBS spectrum

Regulatory constraints imposed on the experimental setup and associated with ablation of uranium dictated the placement of an optical window at the end of a variable length vacuum tube extending from one of the ports of the vacuum chamber. The optical window and vacuum tube serve to contain any possible ablation products within the chamber. At the same time, they allow for filamentation to occur within the vacuum tube after the beam passes through the optical window. As the focusing lens was translated in filament propagation distance resolved measurements, the laser intensity at the optical window varied. This variation results in the dependence of the self-phase modulation of the laser pulse prior to filament formation on the distance of the lens from the optical window.

The standard method to estimate the magnitude of self-phase modulation (SPM) is by calculating the B-integral, defined as

$$B = \frac{2\pi}{\lambda} \int n_2 I(z)\, dz, \tag{S1}$$

where $I(z)$ is the intensity of the laser pulse along the propagation distance z through a material, n_2 is the non-linear index of refraction, and λ is the wavelength. For the 3 mm optical window used in these experiments, the B-integral was calculated to be 0.36 when the optical window was placed immediately after the focusing lens, which suggests that the thin optical window used makes only a small contribution to self focusing. A 10% change of the laser intensity at the optical window increases the B-integral from 0.36 to 0.45. However, as the laser beam is loosely focused to generate laboratory scale filaments, the beam diameter decreases along the propagation distance, which can significantly increase the B-integral. For example, if the window is placed at the location where the beam diameter is reduced by a factor of two from its initial size at the output of the focusing lens, the B integral increases above unity, implying a significant contribution to self-focusing. This self-focusing contribution due to the presence of the optical window results in a change in the position at which filamentation occurs and the overall longitudinal filament intensity distribution. By restricting the distance between the focusing lens and the optical window to $<$30 cm, the maximum variation of the laser intensity and B-integral is limited to $\sim$20% as the distance between the focusing lens and the window is varied. To accomplish this, sections of flexible vacuum tubing

were added to the end of a 1.0 or 2.0 m steel vacuum tube attached to the experiment chamber that maintained the distance between the optical window and focusing lens to be ≤30 cm. In order to elucidate the effect that the variable distance between the focusing lens and optical window has on the LIBS spectra, measurements were performed with the focusing lens at a fixed position (−100 cm) and the optical window placed at four different locations, as shown in Fig. S1. For a distance of ≤30 cm, the variation in the measured intensity of the U_xO_y emission was <10%, which is comparable to the measured variation of the emission (10–15%) at any single location. However, as the distance between the optical window and focusing lens is increased to 60 cm, the emission intensity increases ∼30% over the measured emission intensity when the optical window and focusing lens are separated by 0–10 cm. The increase in the measured emission intensity is due to a change in the longitudinal filament intensity profile as a result of additional SPM due to the presence of an optical window. Thus, we conclude that the variation in the measured LIBS spectra due to the presence of the optical window was relatively small over the 1.8 m filament propagation distance when the distance between the focusing lens and optical window was limited to ≤30 cm, making these results representative of the expected results when no optical window is placed in the path of the focusing laser pulse in air.

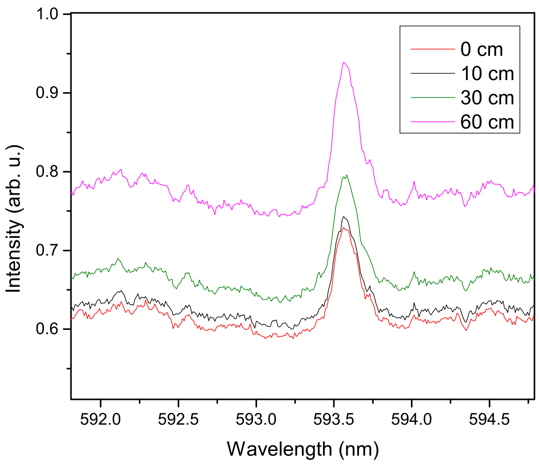

Figure S1: U_xO_y emission dependence on the distance (given in the figure) between the optical window and the focusing lens. The focusing lens was maintained at a fixed position 100 cm before the geometric focus. Ten spectra were averaged using a 2.0 µs gate delay and 5.0 µs gate width for this measurement.

Supplementary Note 2: Identification of the uranium molecular emission

In previous work reported by Heaven *et al.* [S1] and Kaledin *et al.* [S2], the excited UO radicals were produced using a pulsed laser vaporization source that generated a molecular beam of UO from a uranium metal sample, which was cooled to ∼130 K through supersonic expansion. The temperatures and pressures present in these previous studies were orders of magnitude different compared to those found in typical laser produced plasmas. This previous work was used as a starting point for the initial investigation of the UO molecular emission in a laser induced plasma, but it was expected that the intensity and broadening of the measured UO molecular emission within the laser induced plasma would be significantly different.

Heaven *et al.* [S1] identified a number of UO molecular emission features in the 590–600 nm spectral range including the strong Q branch emission centered at 593.6 nm; therefore, this spectral window was chosen to search for the evidence of any UO molecular emission in the measured LIBS spectrum.

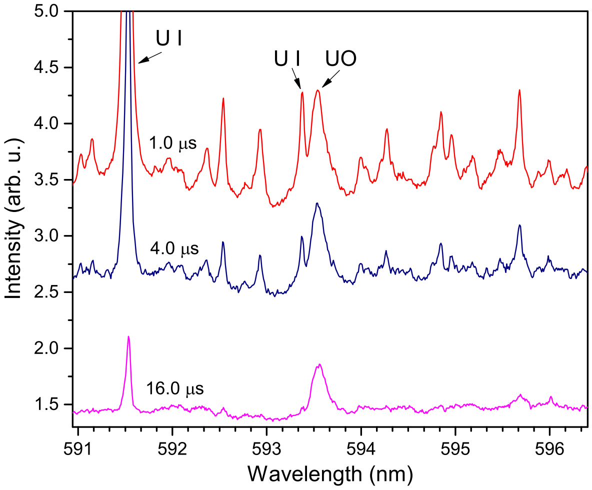

Figure S2: Selected emission spectra from femtosecond laser ablation of uranium under ambient atmospheric conditions for various gate delays and a constant width of 1.0 μs. Ten laser shots were averaged to obtain the measured emission spectrum.

The formation of uranium oxide molecules within the plasma is dependent on the plasma assisted combustion of uranium with oxygen in the ambient atmosphere. A thin oxide layer may be present on the sample surface that could contribute to the formation of uranium oxide in the plasma; however, this layer is readily removed with 5–10 "cleaning" laser shots. It is possible to separate the uranium atomic and uranium molecular emission through the decrease in the atomic emission intensity and increase in the molecular emission intensity at later times in laser induced plasmas. The increase in the uranium molecular emission at later times is due to the decrease of plasma temperature and the expansion of the ablation plume into the ambient atmosphere, which increases the population of molecular radicals. Figure S2 shows selected results of LIBS measurements on the depleted uranium sample under ambient atmospheric pressure as a function of the gate delay for a constant gate width. Immediately apparent in this result is the presence of a strong emission feature centered at 593.57 nm at plasma lifetimes greater than ∼1.0 μs. Review of the Los Alamos Scientific Laboratory Uranium Atlas [S3] and the NIST Atomic Line Database [S4] reveals that this emission line is not associated with either U I or U II. The 593.38 nm U I emission line is observed at delays in the range of 100 ns–4.0 μs, while the 593.57 nm emission feature is first observed after 500 ns and persists up to ∼20 μs. The delay in the observation of the 593.57 nm emission feature combined with the presence of the isolated emission feature at later plasma lifetimes is indicative of it emanating from uranium oxide. At later plasma lifetimes the uranium molecular emission becomes more isolated due to the reduction of the atomic emission; however, the overall emission intensity and signal-to-background ratio was reduced compared to earlier times.

The combination of the long emission lifetime, the emission peaking at later times compared to the atomic emission, and the identification of the uranium monoxide (UO) emission centered at

∼593.6 nm in previous work [S2, S1] suggests that the 593.57 nm emission feature reported here belongs to an oxide of uranium. [S6] It is not possible to further identify the molecular emission feature as UO or another uranium oxide (U_xO_y) from the spectra measured in this work; however, the observed molecular emission line position corresponds to the same line position for a UO emission line identified by Kaledin *et al.* [S2] and Heaven *et al.* [S1].

Supplementary Note 3: Determination of the error associated with the molecular isotope shift of uranium

For the grating, spectrometer, and ICCD detector used in the experimental setup, the pixel size of the ICCD (15 µm) limits the resolution of the measurement. Therefore, the error associated with the measured uranium oxide molecular isotope shift is the magnitude of the spectral width covered by a single pixel on the ICCD detector. A single pixel on the ICCD detector was calculated to cover a spectral width of 0.007 nm.

Supplementary References

[S1] Heaven, M. C., Nicolai, J.-P., Riley, S. J. & Parks, E. K. Rotationally resolved electronic spectra for uranium monoxide. *Chem. Phys. Lett.* **119**, 229–233 (1985).

[S2] Kaledin, L. A. & Heaven, M. C. Electronic spectroscopy of UO. *J. Mol. Spectrosc.* **185**, 1–7 (1997).

[S3] Palmer, B. A., Keller, R. A., & Engleman, J. R. An atlas of uranium emissions intensities in a hollow cathode discharge. *LASL Report LA-8251-MS*, Los Alamos Scientific Laboratory, (1980).

[S4] Kramida, A., Ralchenko, Y., Reader, J. & NIST ASD Team, NIST Atomic Spectra Database (ver. 5.3). [Online], Available: `http://physics.nist.gov/asd`, National Institute of Standards and Technology, Gaithersburg, MD., (2016).

[S5] Dacheng, Z., Xinwen, M., Shulong, W. & Xiaolong, Z. Influence of ambient gas on laser-induced breakdown spectroscopy of uranium metal. *Plasma Sci. Tech.* **17**, 971 (2015).

[S6] Harilal, S. S. & Yeak, J. & Brumfield, B. E. & Suter, J. D. & Phillips, M. C. Dynamics of molecular emission features from nanosecond, femtosecond laser and filament ablation plasmas. *J. Anal. At. Spectrom.* **31**, 1192–1197 (2016).
